# Real-time high-resolution millimeter-wave imaging for in-vivo skin cancer diagnosis

**DOI:** 10.1038/s41598-022-09047-6

**Published:** 2022-03-23

**Authors:** Amir Mirbeik, Robin Ashinoff, Tannya Jong, Allison Aued, Negar Tavassolian

**Affiliations:** 1 Department of Electrical and Computer Engineering, Stevens Institute of Technology, 1 Castle Point Ter, Hoboken, NJ 07030 USA; 2 Department of Dermatologic and Mohs Surgery, Hackensack University Medical Center, Hackensack, NJ 07601 USA

**Keywords:** Biomedical engineering, Electrical and electronic engineering

## Abstract

High-resolution millimeter-wave imaging (HR-MMWI), with its high discrimination contrast and sufficient penetration depth, can potentially provide affordable tissue diagnostic information noninvasively. In this study, we evaluate the application of a real-time system of HR-MMWI for in-vivo skin cancer diagnosis. 136 benign and malignant skin lesions from 71 patients, including melanoma, basal cell carcinoma, squamous cell carcinoma, actinic keratosis, melanocytic nevi, angiokeratoma, dermatofibroma, solar lentigo, and seborrheic keratosis were measured. Lesions were classified using a 3-D principal component analysis followed by five classifiers including linear discriminant analysis (LDA), K-nearest neighbor (KNN) with different K-values, linear and Gaussian support vector machine (LSVM and GSVM) with different margin factors, and multilayer perception (MLP). Our results suggested that the best classification was achieved by using five PCA components followed by MLP with 97% sensitivity and 98% specificity. Our findings establish that real-time millimeter-wave imaging can be used to distinguish malignant tissues from benign skin lesions with high diagnostic accuracy comparable with clinical examination and other methods.

## Introduction

The current general method for diagnosis of skin cancer is through visual inspection by a dermatologist with the aid of a dermatoscope. Dermatologists order biopsy in the cases where cancer is suspected. The visual examination accuracy is highly variable and depends on the level of training and experience of the clinician^1–3^. On the other hand, biopsy and histopathologic examination are not flawless processes. The biopsy procedure is invasive and results in pain, anxiety, scarring, and disfigurement of patients. In addition, histopathological procedure takes as long as several days. More importantly, approximately 15–30 benign lesions are biopsied to diagnose one case of skin cancer^4^. In addition only up to 2% of the samples forwarded to pathology is examined due to tissue processing and sectioning^5^.

Computer-aided diagnostic (CAD) systems for the detection of skin cancer have been developed and are commercially available^6^. Among these are convolutional neural networks (CNN) which have been used for automated classification of skin lesions^7–10^. Although neural networks are trained to attain high sensitivities (> 90%), the achieved specificity rates are low. In real-life clinical settings and when applied to diagnose a broad range of lesions, most dermatologists and CNN systems performed on the same level^11,12^ and even in most situations, more experienced dermatologists outperformed CNN^11^. When set up in clinics, CNN-based systems may produce unacceptably high false-positive rates due to poor specificities, and therefore offer little benefit to clinicians compared with dermatoscopy and their own experience. As such, there is a significant need in the clinic for a device with high diagnostic sensitivity and specificity.

Millimeter-wave imaging (MMWI) is a noninvasive method currently under investigation for skin cancer detection^13–16^. MMWI systems have been traditionally used in various applications such as concealed threat detection^17,18^, security and surveillance^19^, and nondestructive testing (NDT)^20^. A MMWI system can be implemented on a microwave integrated circuit (MMIC) chip, which results in a compact (hand-held) and low-cost system. Microwave integrated circuit technology is currently manufactured at low cost for medium-to-high volume markets such as car radar and communication links. MMWI is capable of detecting biochemical and molecular changes associated with pathological changes^14^. In a large-scale study, we showed that statistically significant contrasts exist between the millimeter-wave dielectric properties of normal skin and two of the most common types of skin cancer, basal cell carcinoma (BCC) and squamous cell carcinoma (SCC)^15^. A total of 101 freshly-excised normal and malignant (BCC and SCC types) skin tissue samples were collected from various body locations of skin cancer patients. Statistically significant differences were observed between the dielectric properties of both BCC and SCC skin cancer tissues and normal skin tissues over the entire range of the millimeter-wave frequencies. The results of this study show that biochemical alterations in malignant tissues, including changes in water molecules, protein, nucleic acid, and glucose concentrations play a major role in the changes in dielectric properties compared to normal tissues.

In another study, we developed a high-resolution millimeter-wave imaging (HR-MMWI) system for ex-vivo experiments with an ultra-wide bandwidth of 98 GHz using the synthetic ultra-wideband millimeter-wave imaging approach^14^. In this system, the ultra-wide imaging bandwidth of 12–110 GHz was synthetically formed by the integration of four adjacent sub-bands and assigning each sub-band to a separate imaging element, i.e., an antenna radiator. Each of the sub-band antennas transmits and receives signals only at its corresponding sub-band. The captured signals are then combined and processed to form the image of the target. Twenty-one non-melanoma skin cancer specimens (comprising of 13 basal cell carcinoma and 8 squamous cell carcinoma specimens) were collected from the Dermatologic, Mohs, and Laser Surgery Center at Hackensack University Medical Center. The specimens were imaged and compared with histopathology. The system was shown to be able to produce high-contrast, three-dimensional (3D) images of the skin. For all the 21 specimens studied, the obtained images correctly identified the locations of the tumors as compared to histological evaluations.

In the above-mentioned ex-vivo study, the performance of the HR-MMWI system was tested on non-melanoma skin cancer tissues. The performance of the imaging technology in detecting melanoma skin cancer as well as differentiating between benign and cancer tissues also requires to be evaluated. This potential to differentiate between benign and cancer tissues will result in a significant decrease in the number of unnecessary biopsies, saving time and effort for dermatologists. It will also reduce patient discomfort and provide significant cost reductions for both the individual patient and the nation’s healthcare system. Therefore in this work, we perform a large-scale in-vivo imaging study on patients having melanoma, non-melanoma, and benign skin lesions. In-vivo measurements were performed at the Dermatologic, Mohs, and Laser Surgery Center at Hackensack University Medical Center.

In the above-mentioned ex-vivo imaging setup, the measurements were not done in real time. This would not cause any issues for ex-vivo experiments. However, in-vivo measurements should be performed in real time to facilitate the procedure for patients as they are required to sit still during measurements. In this work, we reduced the number of sub-band antennas to two from the four antennas previously used in the ex-vivo study. This reduction in the number of sub bands resulted in reducing the imaging time to 20 s, enabling in-vivo imaging of human subjects through a convenient and fast imaging setup.

In addition, in the imaging setup in the ex-vivo experiments, the scanning process was performed automatically using a motorized XY linear stage. At each scanning step, the sub-band antennas were also successively placed in front of the target, transmitted their signals in their respective sub-band ranges, and recorded the backscattered signals. Instead of having sub-band antennas scan the target, it was the target that traveled across an imaging plane. However, in the in-vivo measurements the target could not be placed on stage and as such we developed a motorized XYZ arm which held the sub-band antennas together and enabled 3D scanning of the target.

In this work, a real-time HR-MMWI system is developed for in-vivo skin measurements of more than 136 cases of skin cancer and benign skin lesions. The performance of the HR-MMWI system in differentiating different skin lesions is reported. In particular, the diagnosis of skin lesions with clinical interest, i.e., cancer/precancer versus benign lesions, is concerned here.

## Patients and methods

### Patients

This study was approved by the Institutional Review Board (IRB) at the Research Integrity Office of Hackensack University Medical Center (Hackensack, NJ, USA; Protocol# Pro2019-0219). All of the imaging procedures and experimental protocols were carried out according to the guidelines of the Hackensack University Medical Center's Institutional Review Board (IRB). Patients with age greater than 18 and with clinically suspicious skin lesions were invited to volunteer in this study. Written informed consent was obtained for each patient prior to conducting the measurements. Lesions were not considered for inclusion if they were located at a body site that was inaccessible to the imaging device or had previously been biopsied or excised.

We acquired millimeter-wave images from 136 benign and malignant skin lesions from 71 patients between July 2020 and March 2021. We specifically focused on skin lesions that caused patient and physician concern about skin cancer, including: (i) malignancies and pre-malignancies that require treatment, such as malignant melanoma, squamous cell carcinoma (SCC), basal cell carcinoma (BCC), and actinic keratosis, and (ii) benign conditions that visually mimic skin cancer, such as melanocytic nevi (junctional, compound, and intradermal), angiokeratoma, dermatofibroma, solar lentigo, and seborrheic keratosis. There was a total of 146 lesions including 10 that we invalidated as their reflected millimeter-wave data had obvious spectral dips due to accidental interference and noise. For each lesion, the final diagnosis was established through histopathologic analysis following millimeter-wave measurements and biopsy of the lesion. The final data set consisted of 136 validated lesions from 71 subjects (33 male, 38 female), aged 18 to 94 years (median 61 years). Among these, there were 5 melanomas, 8 SCCs, 10 BCCs, 20 actinic keratoses, and 93 benign conditions. The detailed distributions of the patients and lesions, including the diagnostic subtypes of the lesions and their locations, are provided in Table 1.

**Table 1:** Summary of patients and lesions evaluated by the high-resolution millimeter-wave imaging (HR-MMWI) system.

Localization	Benign lesions (*n* = 93)		Malignant lesions (*n* = 43)	
Non-glabrous common skin	43	(46%)	22	(51%)
Palmoplantar skin	12	(13%)	1	(2%)
Facial skin	28	(30%)	15	(35%)
Hairbearing scalp	10	(11%)	5	(12%)
**Lesion type**				
Melanocytic nevus	22	(24%)		
Seborrheic keratosis	25	(27%)		
Solar lentigo	24	(26%)		
Angioma/angiokeratoma	12	(13%)		
Dermatofibroma	10	(11%)		
Melanoma			**5**	**(12%)**
Basal cell carcinoma			**10**	**(23%)**
Pigmented BCC			5	(12%)
Ulcerated BCC			2	(7%)
Superficial BCC			1	(2%)
Squamous cell carcinoma			**8**	**(19%)**
Actinic keratosis			**20**	**(46%)**

Significant values are in bold.

### Instrumentation

**Figure 1:**
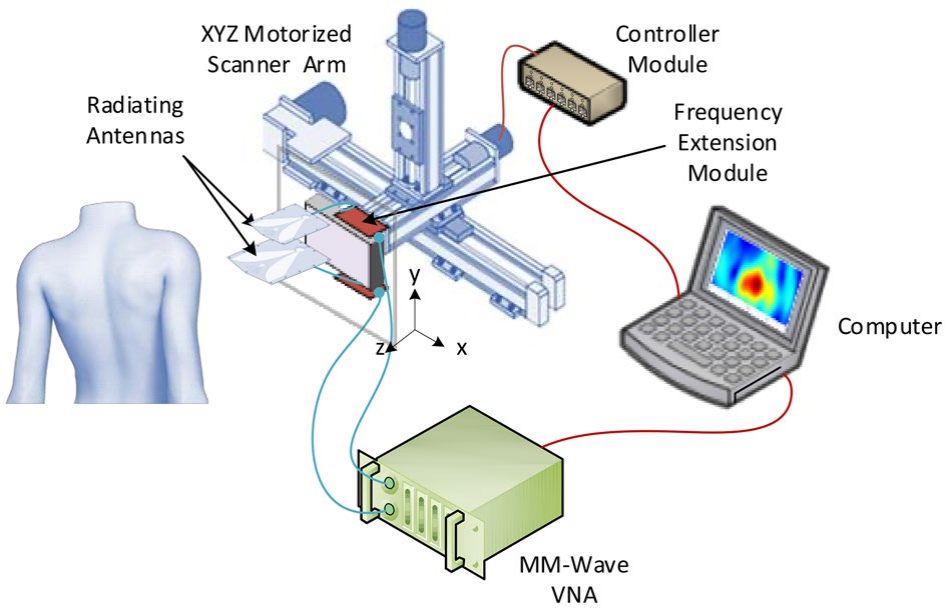
Schematic of the developed ultra-wideband millimeter-wave imaging system for real-time, in-vivo skin cancer imaging, achieving an overall synthetic bandwidth of 98 GHz. At each scanning step, two sub-band antennas are placed in front of the target, transmit their signals in their respective sub-band frequency ranges, and record the backscattered signals.

A HR-MMWI system with a synthetic ultra-wide bandwidth of 98 GHz (12–110 GHz) is used for real-time in-vivo skin lesion demarcation. The schematic diagram of the imaging approach is shown in Fig. 1. In this work, the ultra-wide imaging bandwidth of 12–110 GHz is synthetically formed by the integration of two adjacent sub-bands. For each sub-band, we design an antipodal Vivaldi antenna facilitated by double-ridged substrate-integrated waveguide (SIW). The two similarly-shaped sub-band antennas demonstrate perfect impedance matches (S11 <  − 10 dB) and nearly constant gains of 10 dBi over the frequency sub-bands of 12–51 GHz, and 51–110 GHz respectively, realizing an overall synthetic bandwidth of 98 GHz. At each scanning step, the two sub-band antennas are placed in front of the target, transmit their signals in their respective sub-band frequency ranges, and record the backscattered signals. Imaging is performed in a monostatic manner such that each sub-band antenna scans across M × N positions over the scanning aperture in a rectangular pattern as shown in Fig. 1. It is worth mentioning that in this work, we reduced the number of sub-band antennas to two from the four antennas previously used in the ex-vivo study^14^. In the ex-vivo imaging setup we had previously developed, the measurements were not done in real time. This would not cause any issues for ex-vivo experiments. However, in-vivo measurements should be performed in real time to facilitate the procedure for patients as they are required to sit still during measurements. The reduction in the number of sub-bands resulted in reducing the imaging time to 20 s, enabling in-vivo imaging of human subjects through a convenient and fast imaging setup.

The imaging system platform developed for automatic scanning and data collection is shown in Fig. 1. The scanning process is performed automatically using a motorized XYZ linear arm in conjunction with four drivers, a laptop with LabVIEW programming, and a data acquisition (DAQ) device which generates the digital control signals from a computer. The spacing between consecutive scanning positions is 1.5 mm, which is the maximum spacing that satisfies the Nyquist criterion (~ *λ*/2) for all the frequency sub-bands. The sub-band antennas are placed in the near field of the subject’s skin (ranging from 1 to 3 cm). It is also worth mentioning that the distance between the phase centers of the sub-band antennas and the surface of the target is kept constant. In this way, the signal is transmitted or received as if it were transmitted/received by an equivalent single antenna with a fixed phase center across the entire ultra-wide bandwidth. The sub-band antennas have a field of view (FOV) of at least 4 × 8 mm^2^ on the skin surface (within their near fields). To ensure that the scanning process covers the entire surface of each lesion, each sub-band antenna scans across 8 × 6 positions in a rectangular pattern (aperture plane). Considering the scanning increment of 1.5 mm, a total area of 14.5 × 15.5 mm^2^ is illuminated at each scan.

The collected backscattered signals (S11s) are measured with an Anritsu vector network analyzer (VNA) with W-band modules and imported into the laptop, where signal integration is performed. The integration scheme places the backscattered signals adjacent to each other in the frequency domain to “synthesize” a synthetic ultra-wideband signal covering the whole frequency range of 12–110 GHz. The sub-band antennas are placed at pre-determined positions using the XYZ arm which is connected to the antenna holder. A LabVIEW program controls the stage movements. Finally, the image of the target is formed using a reflectivity function, defined as the ratio of the reflected to incident fields.

To take millimeter-wave measurements, the imaging system is placed in the near-field of the subjects’ skin with no contact. A stand is used to hold subjects’ organs in place while the measurements are taken. The final diagnosis for each measured lesion is established through histopathologic analysis subsequent to millimeter-wave measurements.

Each measurement acquired took 20 s in total. This rapid timing facilitates measurements from multiple sites in a given patient where applicable.

### Millimeter-wave signal processing and image reconstruction

In ultra-wideband imaging, the presence and location of significant backscatters such as a malignant tumor are identified through a reconstruction technique. The main challenge in the image reconstruction procedure is to devise an algorithm which provides high resolutions and suppresses noise and artifacts. Furthermore, in the proposed system which operates with an ultra-wide bandwidth of approximately 100 GHz, the dispersive nature of skin tissues should be considered. Various image reconstruction algorithms have been proposed for microwave and millimeter-wave imaging^21–26^. These techniques are mainly based on the delay-and-sum (DAS) concept in which backscattered signals are time-shifted and summed to create a synthetic focal point of the target. However, these algorithms do not account for dispersive propagation and calculations are performed with the assumption that the target has a constant permittivity. In this work, a reconstruction algorithm in the frequency domain developed previously by the authors is used which takes the dispersive behavior of the target into account^14^.

**Figure 2:**
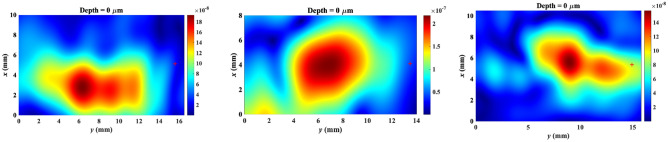
Exemplar millimeter-wave images for different skin pathologies in this study: **(a)** basal cell carcinoma, **(b)** squamous cell carcinoma, and **(c)** actinic keratosis.

Three exemplar millimeter-wave images for different skin pathologies in this study are depicted in Fig. 2. No unique millimeter-wave reflectivity values could be visually assigned to specific skin cancers. Therefore, we use statistical techniques to extract the diagnostic information that is embedded in the millimeter-wave images. As we go deeper into the tissue, the desired reflected signal becomes weaker and the clutter becomes more dominant at the penetration depth. Therefore, the penetration depth in the obtained millimeter-wave images needs to be estimated prior to statistical analyses. As the millimeter-wave image consisted of both normal skin and lesion regions, two depth measures were obtained and then averaged per image. Two spots, one at the central portion and one at the margin of the lesion, were selected for each tissue image. A 30-column range was selected for both regions and the image reflectivity values in each column were summed up and averaged over 30 columns. The average measures were then used as threshold values to determine penetration depth as the point into the tissue up to which the reflectivity remains higher than the thresholds. The images up to the penetration depth were then used for lesion classification.

## Statistical analyses

**Figure 3:**
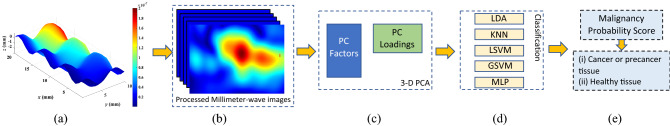
Illustration of the malignancy detection protocol using HR-MMWI. (**a**) Image reconstruction, (**b**) image processing and depth estimation, (**c**) feature extraction using 3D PCA, (**d**) feature classification, and (**e**) cancer detection based on malignancy scores. The details of the steps are provided in “Millimeter-wave signal processing and image reconstruction” and “Statistical analyses”.

The purpose of our classification approach is to produce a binary output: (i) “Tissue sample exhibits characteristics consistent with cancer or precancer,” the lesion should be considered for biopsy; or (ii) “Tissue sample is consistent with healthy tissue,” the lesion does not require biopsy. Figure 3 demonstrates our procedure for malignancy detection using HR-MMWI. First, a multivariate statistical analysis based on principal component analysis (PCA)^27^ is used to automatically determine the most diagnostically significant features. In this work, PCA reduces almost 1000 intensity variables (3-dimensional image cubes with dimensions *x*, *y* and *z*) within the raw tissue images to six PCs, which are linear combinations of the original variables. To obtain the most significant PCs, we calculated the eigenvectors (principal components) of the data covariance matrix. Once eigenvectors are found from the covariance matrix, the next step is to order them by eigenvalue (PC scores), highest to lowest. This gives the principal components in order of significance. Then we used the variance explained criteria approach^28^ to decide on the number of principal components. We set a threshold of 80% and stop when the first *k* components account for a percentage of total variation greater than this threshold. In our case, the first six components account for 83% of the variation. Thus, based on the employed criteria we pick the first six principal components to represent our data set.

The collected PC scores were fed into the classifier model. Several linear and nonlinear classifiers^29^, including Linear Discriminant Analysis (LDA), K-Nearest Neighbor (KNN) with different K-values (K.1, 3, 5, and 7), Linear Support Vector Machine (LSVM), Gaussian SVM (GSVM) with different margin factors (C. 0 to 4 with steps 0.1), and multilayer perception (MLP) were tested using all possible combinations of features. Based on the dataset size, the classifiers were chosen among various machine learning algorithms to classify lesion types with high reliability and robustness. Combining the six obtained PCs and each classifier in various configurations will allow us to have numerous unique discriminators for determining the best values for sensitivity and specificity.

### Three-dimensional principal component analysis (3D PCA)

**Figure 4:**
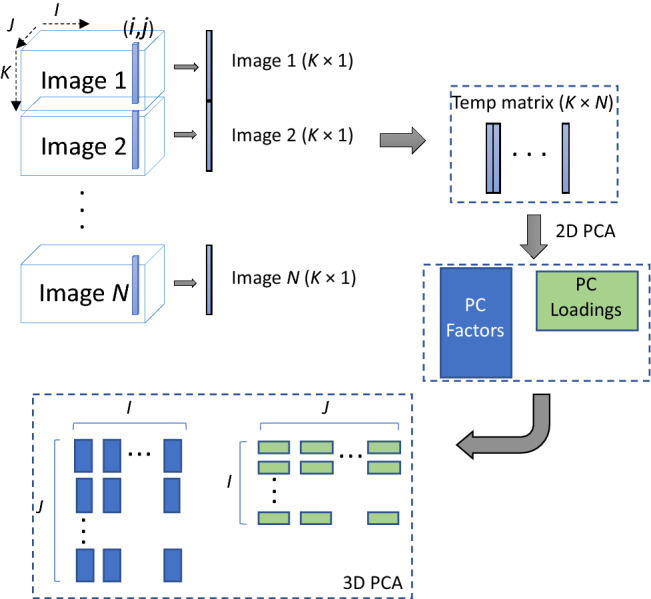
Illustration of data processing using 3D-PCA. Each point (*i*,*j*) throughout the depth in the 3D images is transformed into a temporary 2D structure. A regular PCA decomposition is applied to transformed images of the millimeter-wave image data set. After the number of PCs is selected, the scores and loadings are combined for all points (*i,j*) and separated for each PC.

We employed a new algorithm of three-dimensional principal component analysis (3D-PCA) for exploratory analysis of complete 3D images obtained through millimeter-wave imaging. Figure 4 illustrates the proposed 3-D PCA method. As seen in this figure, each point (*i,j*) throughout the depth of the 3D images is transformed into a temporary 2D structure. A regular PCA decomposition is applied to the 2D structures of the millimeter-wave image data set. The number of PCs (6 in this work) is selected based on the singular values obtained by singular value decomposition (SVD) of the millimeter-wave images^30^, in a similar manner as described by^31^. After the PCs are selected, the scores and loadings are combined for all points (*i,j*) and all PCs.

### Classification results

Due to the small number of subjects, we utilized a k-fold cross validation algorithm with 136 folds^32^; that is, for each classifier 136 combinations of training and test datasets were used. For each PC-classifier analysis, successive single lesional images were left out for “testing,” with the remaining images used for “training.” The leave-one-out cross-validation (LOO-CV) procedure is a computationally expensive procedure to perform; however, it is appropriate when a small dataset is available. The benefit of more evaluated models is a more robust estimate of model performance as each row of data is given an opportunity to represent the entirety of the test dataset. This results in a reliable and unbiased estimate of model performance.

The PC factors and PC loadings of the training data were calculated. The PC factors of the test data were then calculated based on the loadings of the training data and tested against the discrimination model. A posterior probability for malignancy (malignancy score) was calculated for all lesions from the leave-one-out cross-validation (LOO-CV) analysis. Using different discrimination thresholds, different diagnostic sensitivities and corresponding specificities are obtained. (Sensitivity = TP/(TP + FN) and Specificity = TN/(FP + TN), where TP is true positive, TN is true negative, FP is false positive, and FN is false negative). The receiver operating characteristic (ROC) curve, i.e., sensitivity versus “1_specificity” was calculated from the posterior probabilities. When the discrimination between two groups is high, the ROC curve moves toward the left and top boundaries, whereas poor discrimination yields a curve that approaches the diagonal line in the graph. For each PC-classifier combination, the optimum position was determined. Table 2 shows the optimum selection of PCs for each classifier for the highest area under curve (AUC) of the ROC. The best overall performance belongs to the combination of PCs 2 through 6 and the MLP classifier.

**Table 2:** Selection of classifier and PC combinations to achieve the optimum sensitivity and specificity.

Classifier	PC combination [PC_1_PC_2_PC_3_PC_4_PC_5_PC_6_]	Sensitivity	Specificity
LDA	[010101]	0.89 ± 0.05	0.95 ± 0.03
LSVM (C = 1.7)	[111101]	0.92 ± 0.05	0.98 ± 0.01
GSVM (C = 2)	[111111]	0.95 ± 0.04	0.94 ± 0.03
KNN (K = 3)	[101101]	0.95 ± 0.04	0.98 ± 0.02
MLP	[011111]	0.97 ± 0.03	0.98 ± 0.02

The binary numbers in “PC combination” column show if that PC has been used, “1” or not, “0”.

**Figure 5:**
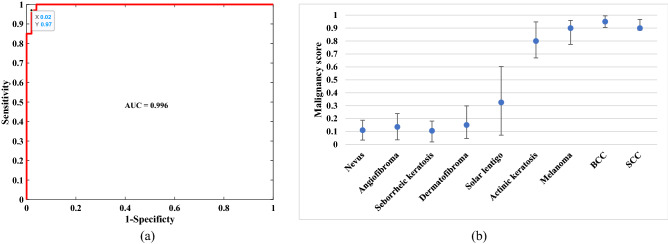
**(a)** The ROC curve of the combination of PCs 2 through 6 with the MLP classifier. **(b)** Blue dots depict the median malignancy probability scores (range 0–1) for major benign and malignant diagnostic categories. Scores closer to 1 indicate a higher probability of malignancy. The upper and lower bounds indicate the standard deviation.

The ROC curve of the best combination is shown in Fig. 5a. It shows a sensitivity and specificity of 97.0% and 98.0% respectively. The AUC of the ROC is 0.996. Boxplots in Fig. 5b show the distribution of malignancy scores (posterior probability of the test lesion for skin cancer) in relation to diagnostic categories. With the a priori malignancy cutoff at > 0.5, the percentage of correct classifications in malignant lesions was 95% in AK, 100% in melanoma, 100% in basal cell carcinomas (BCCs), and 88% in squamous cell carcinomas (SCCs). In the benign lesions, the percentage of correct classifications was 95% in nevi, 100% in angioma/angiokeratoma, 96% in seborrheic keratoses, 100% in dermatofibroma, and 92% in solar lentigo.

In order to evaluate the classifier performance for larger datasets, we used an approach in which the expected classifier performance for large datasets is extrapolated based on error rates calculated from smaller datasets^33^. This approach employs random repeated sampling (RRS) in conjunction with a cross-validation sampling strategy. We obtained the extrapolated error rates for each classifier with the PCs combination that resulted in the best performance. We compared the extrapolated error rates among the 5 classifiers. It was determined that both GSVM and MLP classifiers yielded lower error rates than that of the other three classifiers and are therefore more robust against the data size.

To evaluate the reproducibility of the millimeter-wave measurements, we conducted an independent study in which reflected data were repeatedly taken from the same sites of ten different locations of normal skin. Variability in the millimeter-wave frequency shifts (corresponding to the abscissas) from the same skin site was negligible. This confirms the relative stability of spectral minimum positions. The successive variances at each millimeter-wave frequency were also calculated and were able to define the frequencies at which the spectra reached a maximum variance for all the repeat-matched reflectivity measurements. This variance reached a maximum of 5%.

We did a sub-analysis for lesions from only the head in terms of discriminating cancerous and precancerous lesions from benign lesions. The AUC of this ROC curve is 0.998, which is close to the AUC obtained for all body sites; these two ROC curves were not statistically different (P. 0.5531).

## Discussion

The aim of this study was to evaluate the performance of the HR-MMWI system in discriminating skin cancer from benign skin lesions. Statistical techniques were used to extract the diagnostic information embedded within the millimeter-wave signals. Our HR-MMWI system was designed to operate in frequency range of 12–110 GHz as this range contains a dense cluster of peaks^15^.

Several noninvasive imaging approaches have been developed for the diagnosis of skin cancer and their differentiation from benign lesions. Their clinical utility however, is limited as each provides information only under certain conditions.

The technologies currently evaluated for skin cancer detection are summarized in Table 3. Most of these techniques have been created specifically for skin evaluation, although some can be applied to other areas of medical imaging and medicine. Reflectance confocal microscopy (RCM) uses near-infrared laser for in-vivo imaging of thin sections of skin. An algorithm for diagnosing skin cancer based on RCM features was 100% sensitive and 88.5% specific when tested on nearly 800 lesions^34,35^. Optical coherence tomography (OCT) employs projecting infrared light onto the skin to create an image based on the sum of light refractions of various skin structures with different optical properties. In a study, OCT had a sensitivity and specificity for BCC diagnosis of 87% and 80%, respectively^36^. OCT had the highest accuracy (87.4%) when used in conjunction with dermatoscopy^37^.

**Table 3:** Sensitivities and specificities of skin cancer detection techniques.

Technology	Primary type of skin cancer	Sensitivity range (%)	Specificity range (%)
RCM	Melanoma	93	78
OCT	BCC	89–96	60–78
RCM + OCT	BCC	100	75
MSDSLA	Melanoma		
Melafind		94–100	6–40
SIAscopy		83–85	80–81
EIS	Non-melanoma Melanoma	98–100 97–99	34–55
RS	Non-melanoma Melanoma	69–81 93	85–89 96
HR-MMWI (this work)	Non-melanoma Melanoma	97	98

*RCM* reflectance confocal microscopy, *OCT* optical coherence tomography, *MSDSLA* multispectral digital skin lesion analysis, *EIS* electrical impedance spectroscopy, *RS* Raman spectroscopy, *HR-MMWI* ultra-high-resolution millimeter-wave imaging.

In multispectral digital skin lesion analysis (MSDSLA), visible and infrared light are employed to image pigmented lesions suspicious for melanoma with the aid of computer algorithms which determine the malignancy likelihood. The two most studied MSDSLA techniques include MelaFind and Spectrophotometric Intracutaneous Analysis (SIAscopy). The biopsy ratio approximately ranged from 7.6:1 to 10.8:1 for identifying melanoma using MelaFind^38^. Another study on the performance of SIAscopy found a sensitivity of 82.7% and specificity of 80.1% when used to identify 348 pigmented lesions^39^.

The basis of electrical impedance spectroscopy (EIS) is that the malignant alteration in cells changes the electrical impedance. In^40^, the diagnostic accuracy of clinical assessment on 449 skin lesions is compared to when EIS is employed. The addition of EIS increased the melanoma diagnosis sensitivity from 81 to 98% and decreased the specificity from 84 to 55%.

The Raman effect principle specifies the small change in incident photon energy and a scattered photon. The color of the scattered photon slightly changes as a result of the shift in the energy. Raman spectroscopy (RS) of the skin outlines these shifts over a spectrum to generate the tissue molecular characteristic. The most recent review of Raman spectroscopy for skin was conducted in 2018 and includes 12 in-vivo studies with 2641 Raman spectra^41^. Their results are as follows: sensitivity of 69% and specificity of 85% for BCC, of 81% and specificity of 89% for SCC, and sensitivity of 93% and specificity of 96% for melanoma.

The integration of non-invasive techniques may also be beneficial. For instance, the integration of OCT and RCM into a combined system may provide comprehensive three-dimensional, real-time imaging to enhance skin cancer diagnosis^42^. Sahu et al. found in a pilot study that when OCT and RCM were combined into a single probe, the imaging techniques complemented each other for basal cell carcinoma (BCC) diagnosis^42^. When the combined device was used on 85 lesions from 55 patients, diagnosis of BCC was achieved with 100% sensitivity and 75% specificity.

Another combination of two skin imaging methods—multispectral and autofluorescence—has been applied to skin cancer diagnostics. In a reference^43^, multispectral fluorescence lifetime imaging (FLIm) dermoscopy system was designed and used to image 38 patients with diagnosed nodular basal cell carcinoma (nBCC) lesions. Statistical classifiers for discriminating BCC from healthy tissue showed an AUC of the ROC performance of 0.82.

Although there have been several promising imaging approaches in the field of skin cancer detection, various translational issues still need to be solved. Cost is the main barrier to widespread implementation of skin cancer detection devices. An RCM machine is priced at about $100,000, and MelaFind installation costs up to $10,000 in addition to annual fees^44,45^. Another barrier for several technologies is the requirement for experienced and/or trained professionals. Finally, lack of efficiency, low specificity, and limitations on anatomic sites are issues that need to be considered. As such, only a limited number of dermatologists are currently using new technologies. This number could increase as the technologies, their diagnostic accuracies, and their limitations are better understood^46^.

Tissues have intrinsic characteristics based on their density, size, and shape of tissue microstructures; the absorption characteristics derived from water and biochemical contents of the tissue. These characteristics are modified during tumor development. Methods such as HR-MMWI that can uniquely identify these characteristics hold promise for providing diagnostic value. Our results are promising compared other diagnostic aids. We showed that real-time HR-MMWI could distinguish skin cancer and precancer lesions from benign tissues with an AUC of the ROC curve of 0.996, and sensitivity and specificity of 97% and 98% respectively. The studies mentioned above as well as our study have assessed specific diagnostic methods independently. In a clinical setting, the final diagnosis of any suspect skin lesion is in fact considered by collectively using all the available data and evidence. This is heavily influenced by the insight and experience of the clinician. One significant advantage of the HR-MMWI system compared to other technologies is its ability in acquiring images within seconds or less. In other techniques, the objectives are primarily to differentiate skin cancers from normal skin.

The functionality of the HR-MMWI system can be further enhanced by integrating all the imaging antennas along with their corresponding circuits in a single framework by employing the microwave integrated circuit technology. A welcome advantage of the short wavelengths of millimeter waves is the downscaling, in particular, of sensors and antenna interfaces, which is beneficial when designing handheld or point-of-care devices. Consequently, instead of a benchtop HR-MMWI system, a handheld configuration can be designed, conveniently providing in-vivo images of the skin tissue at a very low manufacturing cost. This capability will allow dermatologists and dermatologic surgeons to obtain histopathological-like images of cancer tissues prior to biopsy or subsequent to tumor excision, enabling immediate detection and removal of residual tumors. A hand-held, real-time imaging tool with a comparable price to a dermatoscope will have a huge value as it helps dermatologists visit more patients in different rooms or at different clinics.

Overall, the results of this work support the use of HR-MMWI in assisting skin cancer detection at the clinical interest level, i.e. cancer/precancer versus benign. Considering the specificity rates obtained in this study, MMWI could reduce the number of unnecessary biopsies by more than 50%. HR-MMWI is an affordable technique and requires less extensive training and user expertise. We envision that the classification of a given lesion can be performed in less than 20 s, making this approach a novel clinical contribution to detecting and managing skin cancer, the most common cancer type.

### Limitation

The performance of the HR-MMWI was not uniform across classes. This is due to the relatively low frequency of all disease categories in the training set.

## Conclusion and future work

We have studied skin cancers and a broad range of benign skin lesions using real-time in-vivo ultra-high-resolution millimeter-wave imaging (HR-MMWI) with imaging time of less than 20 s per lesion. Statistical analysis shows that HR-MMWI can distinguish cancer and precancer lesions from benign disorders with 97% sensitivity and 98% specificity. The results indicate that HR-MMWI can be used to distinguish between malignant from benign skin lesions with high diagnostic accuracy comparable with clinical examination and other methods.

Although the capability of the HR-MMWI technology in differentiating different types of skin cancer from benign tissues and normal skin has been verified, its capability to identify tumor margins pre- or intra-operatively needs to be verified as well. In our future research, a novel method will be developed for tumor margin identification in 3-D millimeter-wave skin imaging. The images will then be segmented into tumor and normal areas using the tumor margins. The effectiveness of our algorithm will be evaluated using numerical and realistic skin phantoms. In addition, all the imaging sub-band antennas with their circuits will be integrated in a single chip by employing the monolithic microwave integrated circuit (MMIC) technology. This will result in a compact (handheld) and real-time imager at a very low manufacturing cost.
